# Rationale and Design of a Genetic Study on Cardiometabolic Risk Factors: Protocol for the Tehran Cardiometabolic Genetic Study (TCGS)

**DOI:** 10.2196/resprot.6050

**Published:** 2017-02-23

**Authors:** Maryam S Daneshpour, Mohammad-Sadegh Fallah, Bahareh Sedaghati-Khayat, Kamran Guity, Davood Khalili, Mehdi Hedayati, Ahmad Ebrahimi, Farhad Hajsheikholeslami, Parvin Mirmiran, Fahimeh Ramezani Tehrani, Amir-Abbas Momenan, Arash Ghanbarian, Atieh Amouzegar, Parisa Amiri, Fereidoun Azizi

**Affiliations:** ^1^ Cellular and Molecular Endocrine Research Center Research Institute for Endocrine Sciences Shahid Beheshti University of Medical Sciences Tehran Islamic Republic Of Iran; ^2^ Prevention Of Metabolic Disorders Research Center Research Institute For Endocrine Sciences Shahid Beheshti University Of Medical Sciences Tehran Islamic Republic Of Iran; ^3^ Nutrition And Endocrine Research Center Research Institute For Endocrine Sciences Shahid Beheshti University Of Medical Sciences Tehran Islamic Republic Of Iran; ^4^ Reproductive Endocrinology Research Center Research Institute For Endocrine Sciences Shahid Beheshti University Of Medical Sciences Tehran Islamic Republic Of Iran; ^5^ Endocrine Research Center Research Institute for Endocrine Sciences Shahid Beheshti University of Medical Sciences Tehran Islamic Republic Of Iran; ^6^ Research Center For Social Determinants Of Endocrine Health Research Institute For Endocrine Sciences Shahid Beheshti University Of Medical Sciences Tehran Islamic Republic Of Iran

**Keywords:** cardiometabolic disorders, cardiovascular, genetic, genome-wide association study

## Abstract

**Background:**

Cardiometabolic risk factors comprise cardiovascular diseases and/or diabetes, and need to be evaluated in different fields.

**Objective:**

The primary aim of the Tehran Cardiometabolic Genetic Study (TCGS) is to create a comprehensive genome-wide database of at least 16,000 Tehranians, who are participants of the ongoing Tehran Lipid and Glucose Study (TLGS) cohort.

**Methods:**

TCGS was designed in collaboration with the Research Institute for Endocrine Sciences and the genetic company deCODE. Participants had already been followed for over a 20-year period for major cardiometabolic-related health events including myocardial infarction, stroke, diabetes mellitus, hypertension, obesity, hyperlipidemia, and familial hypercholesterolemia.

**Results:**

The TCGS cohort described here comprises 17,186 (86.3%) of the 19,905 TLGS participants who provided a baseline blood sample that was adequate for plasma and deoxyribonucleic acid analysis. This study is comprised of 849 individuals and 3109 families with at least one member having genotype information. Finally, 5977 males and 7422 females with the total genotyping rate of 0.9854 were genotyped with HumanOmniExpress-24-v1-0 bead chips (containing 649,932 single-nucleotide polymorphism loci with an average mean distance of 4 kilobases).

**Conclusions:**

Investigations conducted within the TCGS will seek to identify relevant patterns of genetic polymorphisms that could be related to cardiometabolic risk factors in participants from Tehran. By linking genome-wide data to the existing databank of TLGS participants, which includes comprehensive behavioral, biochemical, and clinical data on each participant since cohort inception in 1999, the TCGS will also allow exploration of gene-gene and gene-environment interactions as they relate to disease status.

## Introduction

### Noncommunicable Diseases and Their Health Burden

A noncommunicable disease (NCD) is a medical condition or disease that can be defined as nontransmissible among people. NCDs refer to chronic diseases or conditions in which progression is slow and may last for a long period, or may be lifelong. NCDs, being life threatening conditions, drain economic resources, increase mortality and morbidity in society, and have negative effects on development and economic growth [1]. Claiming 63% of all deaths worldwide, these diseases are currently the world’s most common killer [2], and 80% of these deaths now occur in low- and middle-income countries [3]. This finding also shows that half of those who die of chronic NCDs are in the prime of their productive years, and thus the disability imposed and the lives lost are also endangering industrial competitiveness across borders [3]. Therefore, managing and preventing NCDs are national priorities for several countries, as well as the World Health Organization [4].

The term cardiometabolic is intended to cover cardiovascular and metabolic diseases including diabetes, obesity-related traits, and biomarkers known to be associated with the risk of cardiovascular disease [5,6]. Genetically predisposed individuals have inherent risks independent of environmental factors. Nevertheless, at-risk individuals can adopt healthy lifestyles so that other factors do not augment their risk for the disease. Genetic susceptibility implies an increased likelihood for an individual developing a particular disease, based on his/her genetic makeup. Such predisposition results from specific genetic variations that are often inherited from an individual’s parents [7]. Accumulating evidence supports the role of genetic changes in major NCDs including cancer, diabetes, cardiovascular diseases, mental health, and asthma [8].

With advances in our understanding of the genetic basis of human disease, it has become apparent that the underlying causes of many chronic disorders are multifactorial and involve the complex interplay between acquired and inherited risk factors [9,10]. In most cases copresence or interactions between several molecular changes across the genomic landscape will work together to make one prone or resistant to a given condition. With the advent of genome-wide scanning technologies, it is now possible to obtain information on most of the common genetic variations in individual patients. Obtaining this genome-wide information in well-characterized patient groups (with or without risks for disease) is a critical step in moving toward a genome-based practice of medicine that not only provides insights into the root causes of disease, but also forms the basis for discovery of new and specific targets for drug therapies. Genome-based medicine may also fulfill the promise of personalized medicine and provide the means to implement patient-specific preventive programs years in advance of clinical symptoms [11,12].

A favored analytic approach for such discovery is the genome-wide association study (GWAS), in which genetic variation across the human genome is compared between patients with different disease states or different risk-factor profiles. Success in GWAS requires a comprehensive knowledge of genome-wide variation and linkage disequilibrium patterns, the availability of dense genotyping chip sets containing several hundred thousand single-nucleotide polymorphisms (SNPs), and the availability of large, well-phenotyped patient populations [13].

A potentially more powerful approach than GWAS is the large-scale prospective cohort study, in which initially healthy individuals are followed over long periods of time and assessed for disease development, and all members of the cohort undergo comprehensive genotyping. Such prospective cohort studies have the advantage of avoiding bias in the selection of case and control subjects, and enable simultaneous evaluation of many environmental exposures and potential disease states in an epidemiologically efficient manner. In the present study, we aimed to analyze the genomic chip type of all Tehran Cardiometabolic Genetic Study (TCGS) participants to estimate the genetic pattern of this population. After deep sequencing to generate the Iranian reference panel, the imputation will be performed for each trait, and associations will be analyzed.

### Knowledge Gap

We aimed to undertake a GWAS to evaluate genetic patterns for cardiometabolic risk factors in a Tehranian population, and compare these patterns to other reference genetic databases. This is one of the first studies of its kind in the Middle East, and addresses the knowledge gap on allele frequencies, genetic associations, and the role of consanguineous marriage among Iranian families.

## Methods

### Objectives and Study Variables

The primary objectives of this study are: (1) to measure and analyze deoxyribonucleic acid (DNA) sequence variations from across the Tehranian human genome, in an effort to identify genetic patterns for cardiometabolic risk factors in the population; and (2) to conduct a GWAS to identify genetic variants that are associated with cardiometabolic disorders, use genetic risk factors to predict those at risk, and identify the biological underpinnings of cardiometabolic disease susceptibility to develop new prevention and treatment strategies. The secondary objectives of this study are: (1) to determine inheritable genetic risk factors among families of the TCGS with heritability analyses; and (2) to compare the different genetic patterns that contribute to cardiometabolic outcomes among this population, in different case and control groups.

### Overall Study Design

The TCGS is a prospective family-based GWAS cohort that has been followed since 1999 within the Tehran Lipid and Glucose Study (TLGS), which includes over 15,000 initially healthy subjects >3 years old, who have already been followed for more than 20 years. Participants have been followed for the development of common disorders such as myocardial infarction, stroke, diabetes mellitus, hypertension, obesity, familial hypercholesterolemia, hyperlipidemia, habitation (eg, smoking and physical activity), and biochemical factors (ie, high cholesterol, low high-density lipoproteins, high triglycerides).

The concept of designing a genomic bank from TLGS samples was first presented to the Endocrine Research Center (ERC) and the Iranian molecular medicine network, and was funded by FA and MSD (grant number 147, 2004; grant number 265, 2008). In 2008, a project determining pedigrees according to genetic relationships was funded by ERC (grant number 321), with MSD and AAM as principal investigators. Funding of the main study began in June 2012 with an agreement between the Research Institute for Endocrine Sciences (RIES) and the deCODE genetic company (Reykjavik, Iceland), with FA and MSD as primary investigators. The final protocol for the genetic study was written by FA, MSD, MSF, and DK, and was submitted to the Ministry of Health and Medical Education in August 2012. The protocol was approved by the National Committee for Ethics in Biomedical Research in December 2012.

In this paper, we describe the TCGS (and its parent TLGS) from the perspectives of cohort assembly, follow-up, endpoint validation, baseline plasma phenotyping, DNA extraction, genotyping, participant confidentiality, power, and sample size, and discuss the TCGS in the context of other ongoing GWASs being performed in related areas. The study is organized into 5 phases: (1) cohort assembly and prospective follow-up, (2) genomic sample extraction, (3) phenotype and outcome gathering, (4) chip typing and genotype analysis, and (5) drawing family trees.

#### Cohort Assembly and Prospective Follow-Up

**Figure 1:**
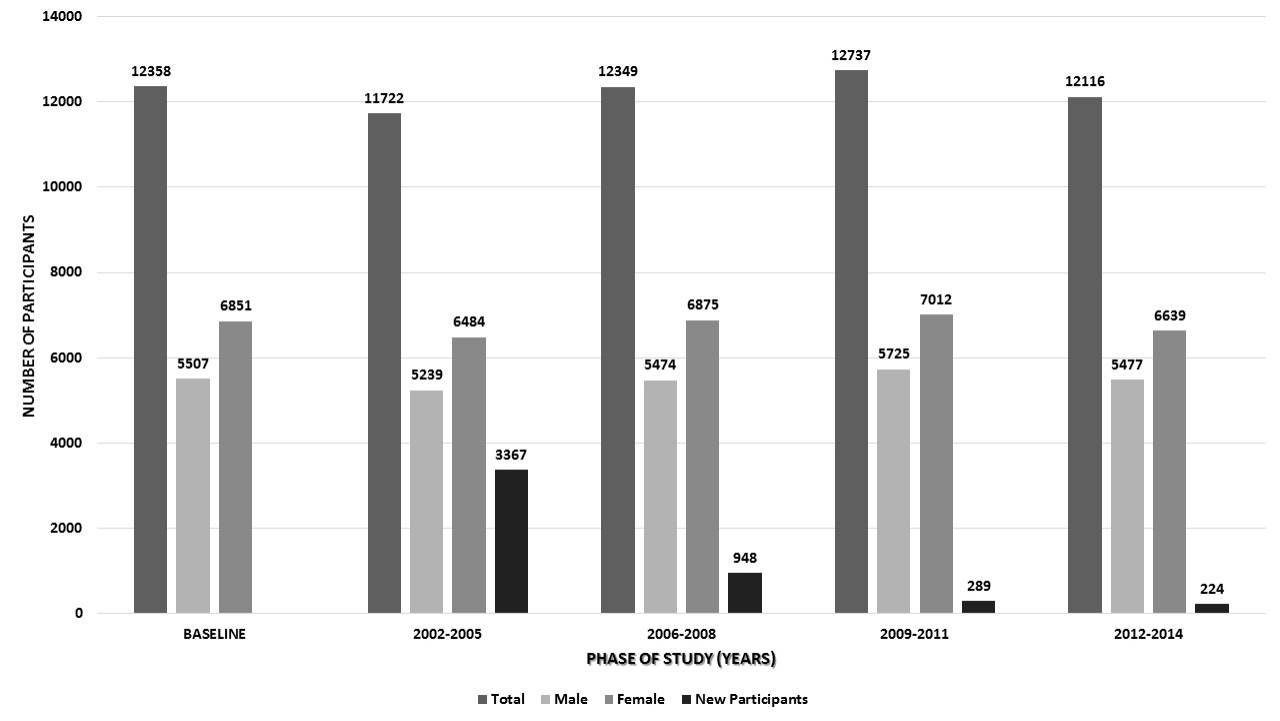
Cohort participation and gender distribution of the study phases.

All members of the TCGS cohort were participants in the TLGS who had provided an adequate baseline blood sample for plasma and DNA analysis, and had given written consent for blood-based analyses and long-term follow-up. The TLGS is a long-term integrated community-based program for the prevention of NCDs by developing of a healthy lifestyle and reducing NCD risk factors. The study began in 1999, and will be continued for at least 20 years. A primary survey was performed to collect baseline data for 15,005 Iranian individuals born between 1912 and 2008, and was selected from cohorts at three medical heath centers [14]. The study designs for TLGS and TCGS were approved by the Institutional Review Board of the RIES, Shahid Beheshti University of Medical Sciences, and all participants provided written informed consent for genetic analyses. The presence of participants in each phase of study shown in Figure 1.

#### Genomic Sample Extraction

At the TLGS sample collection center, a blood sample was drawn into vacationer tubes from all study participants, between 7:00 and 9:00 a.m. after 12-14 hours of overnight fasting. Two blood samples were taken in a sitting position per standard protocol. The blood collected in ethylenediaminetetraacetic acid-containing test tubes was used to obtain DNA samples, which were immediately sent to the genomic laboratory. All TLGS samples were recoded as a genomic sample that connected to the TLGS code in a database. DNA samples were extracted from buffy-coat samples from each participant using a proteinase K/salting out standard method [15]. The quality and quantity of extracted DNA were evaluated. A Thermo Scientific NanoDrop 1000 Spectrophotometer was used to qualify samples, which were aliquoted into 1.5 milliliter tubes and stored in -80°C ultra-freezers for future studies.

#### Phenotype and Outcome Gathering

A broad range of epidemiologic data related to behavioral, dietary, and environmental risk exposures were received from TLGS as a parent study to TCGS. Each TCGS participant also provided a baseline blood sample that had already been evaluated for multiple disease biomarkers, including total cholesterol, high-density lipoprotein-C, low-density lipoprotein-C, triglycerides, fasting plasma glucose, fasting insulin, and creatinine. Individuals with genomic samples that had full familial information will be included in the TCGS, and cardiometabolic risk factors will be measured and defined in this dataset. The specified families with multiple signs of these risk factors will be followed-up.

#### Chip Typing and Genotype Analysis

Portions of DNA samples were genotyped with HumanOmniExpress-24-v1-0 bead chips (containing 649,932 SNP loci with an average mean distance of 4 kilobases) at the deCODE genetics company (Reykjavik, Iceland) according the manufacturer’s specifications (Illumina Inc., San Diego, CA).

#### Drawing Family Trees

At this step, TCGS focuses on drawing all genetic relationships. On the initial day of examination, the TLGS participants were interviewed to obtain demographic data and relationship information, or update existing data. Genealogy data was drawn in Genepro (V 2.0.1.6) and checked by Family-Based Association Tests (FBAT-Toolkid V 1.7.3) [16]. Family data, pedigree information, phenotype, and genotype data were stored, manipulated, and error-checked using the genetic data management system (Progeny Clinical Version 7) from Progeny Software (Progeny Software LLC, Delray Beach, FL). The name of the father was checked for all individuals, and if duplicate names were identified their genetic relationship was checked with their genotype information. If the genetic relationship was confirmed with genotype information, the family tree joint was merged to generate a mega family.

### Genetic Association

The present study aims to genotype TLGS participants. Genotyping will be based on a standard SNP array platform suitable for performing a GWAS comprising tag SNPs from all three HapMap phases, and has been strategically selected to capture the greatest amount of common variation and drive the discovery of novel associations with traits and diseases. Incidence rates of cardiometabolic risk factors (ie, diabetes mellitus, angina pectoris, myocardial infarction, hypertension) will be estimated, and adjusted for age and sex. Other variables, such as education level, physical activity, smoking habits, nutritional habits, and drug use will be use as covariables. Relative risks for different factors and their 95% CIs will be reported. Longitudinal linkage analyses will be performed for families, and selected genetic regions will be examining via association analyses. By using common genetic polymorphisms with well-understood effects on exposure patterns, a causal effect from observational data in the presence of confounding factors will be estimated by Mendelian randomization. The most important phenotypes in this project are cardiometabolic risk factors, including diabetes, cardiovascular disease, obesity, and metabolic syndrome. All phenotypes will be analyzed using a case-control and familial based design, and the related genetic regions will be analyzed and replicated in other populations. An Iranian reference panel will be designed and imputed based on a reference panel that will be performed to obtain information about a higher number of variants.

Multifactoriality is expected to play a pivotal role, and this study is currently focused on collecting information on the genetic and environmental factors that potentially influence cardiometabolic diseases. For the gene-gene and gene-environment interaction analyses, it will be assumed that genetics may influence disease risk either directly or via environment effects. The genetic loci can have either independent or epistatic effects, so the model will be a multilogistic model. However, if nonparametric gene-gene interaction is desired, semiparametric regression and least square kernel machines will be used occasionally.

According to the outcome and phenotype information, different ethnicities, and homozygosity fraction, samples will be selected for whole genome analysis to make an Iranian reference panel in deCODE (Reykjavik, Iceland). After making the reference panel, the genotype data set will be imputed to larger data set.

### Epigenetic Analyses

To explore the association between the methylation pattern of cytosine phosphate guanine (CpG) islands in the regulatory regions of determined genes and main outcomes (ie, obesity, cardiovascular diseases), we will examine the methylation alterations in the CpG regions of determined genes among TCGS participants using methylation-specific polymerase chain reaction, bisulfite-sequencing polymerase chain reaction, and epigenome-wide analysis techniques. Identifying epigenetic modifications associated with cardiometabolic risk factors, including DNA methylation variation, may point to genomic pathways that are dysregulated in numerous conditions. The Illumina Bead Chip array will be use to assay DNA methylation in leukocyte DNA obtained from TCGS participants. Mixed-effects regression models will be used to test the association of methylation beta value with cardiometabolic risk factor changes, adjusting for batch effects and potential confounders. Association analyses of the DNA methylation patterns and TCGS phenotypes will subsequently be performed.

### Sample Size and Power

Sample size and power calculation were estimated according to the TLGS [14] criteria, with the total TCGS duration of 20 years. Due to the wide scope of the project and variety of measured variables, different types of analyses will be performed, depending on the type of report, or the variables and outcomes. Continuous data will be expressed as their measures of location and spread in total, and in subgroups. For qualitative and categorized continuous variables, percentages and frequency distributions will be reported.

Normality of distribution of the continuous variables will be examined using histograms, measures of skewness and kurtosis, Kolmogrove-Smirnove tests, and Chi-square tests. When log-transformed values are used, geometric means will be computed. Percentiles will be used to describe the high or low values of the skewed variables. When assumptions of the parametric statistical methods are not met, nonparametric methods of their counterparts will be used. These methods will also be used for analyses of variables that are not originally continuous, such as attitudes or quality of life.

## Results

### Participant Confidentiality

Participant confidentiality in the TCGS is maintained throughout all aspects of the study, as is the case in the TLGS. Investigators within the TCGS have no access to any direct patient identification information; these data are held confidentially by staff members of the TLGS, who are involved in patient contact and follow-up, but not in any data analysis or interpretation. Separate data files are kept for participants’ clinical covariate and endpoint data, plasma phenotyping data, and genomic data. Blood samples sent to the plasma phenotyping laboratory and the genetic laboratories are labeled only with a sample identification number that cannot be tracked by laboratory personnel to any patient identification variables, or to any clinical covariate data. All TCGS data included in the TLGS are maintained on a separate and fully protected computer system that is isolated and distinct from computing systems used for the parent study. A unique and fully distinct participant identification number is used in the TCGS, making direct linkage to the TLGS impossible for scientific investigators.

### Participants’ DNA Information

Since the establishment of the genomic bank in 2004, some subjects only participated in Phase 1 of the study (1999-2002), and their data has been excluded from this study. The TCGS has a genomic bank with over 16,000 samples. The TCGS cohort described here comprises 17,186 (86.34%) of the 19,905 TLGS participants, who provided baseline blood samples that were adequate for plasma and DNA analyses (Multimedia Appendix 1). This study is comprised of 849 individuals and 3109 families with at least one member who had genotype information.

### Familial Information

The pedigrees were drawn (based on questionnaire information) for all biologically related TCGS participants. Total family data of Tehranian residents (consisting of 3109 families) were collected; 849 unrelated persons, adopted persons, childless bride and groom couples, and individuals who lacked information were marked as *independent persons*. The mean pedigree size in the study population was 6.76 (range 3-52). Placeholding individuals were imputed when necessary to reflect kinship relations among participants, and a total of 6158 individuals were added as virtual persons that were recorded by special codes for further tracking. For example, parents who had not participated in the TLGS were added during the drawing of pedigrees to identify sibships in the TCGS.

### Demographic, Clinical, and Biochemical Information

Demographic, clinical, and biochemical information of participants (categorized by year) in each period of study are presented in Multimedia Appendix 2 and Multimedia Appendix 3. Most the study population was in the range of 31-50 years old. Phenotype frequencies of hypertension, dyslipidemia, diabetes, obesity, and metabolic syndrome were described in adults, and being overweight was the most frequent among them (Multimedia Appendix 4).

### Genotype Quality Control Analysis

PLINK program (V 1.07) and R statistic (V 3.2) were used for quality control procedures. 13,894 samples were arrayed, consisting of 6274 males and 7614 females, with the total genotyping rate of 0.9774.

To increase the power of the analyses, some markers (and some samples) were removed from TCGS database after quality control [17]. In the first step of sample quality control, individuals with discordant sex information were identified. In total, 15,031 X chromosome variants were detected in 397 samples, which were removed from analyses. To evaluate heterozygosity among genotyped samples, plots were drawn and the observed heterozygosity rate per individual was examined to decide reasonable thresholds for excluding individuals based on elevated missing heterozygosity or extreme heterozygosity (Multimedia Appendix 5). In this step, 243 individuals were excluded with a genotype failure rate (>0.03) and/or heterozygosity rate >3 standard deviations from the mean. To eliminate the genetic influence of sample contamination, duplications and cryptic first-degree relative sibling pairs, genome-wide average identity by state (IBS)/identity by descent (IBD) values were calculated for each pair of individuals in the present GWAS data set using pruned SNPs (649,932). To check kinship relationships, IBDs were calculated and all relationships were confirmed.

To find individuals with large-scale differences in ancestry, a principal component analysis was conducted and 47 subjects were removed (Multimedia Appendix 6). For marker quality control, those with a minor allele frequency less than 0.01 (n=24,860) and genotype failure rate less than 0.95 (n=2620) were removed. After excluding markers that did not satisfy the Hardy Weinberg Equilibrium (n=14,032, *P*<.001), 608,420 remained for analyses. Multimedia Appendix 7 shows the distribution of SNP call rate after the cleaning process. In summary, the clean data set included 13,399 individuals consisting of 5977 males and 7422 females, with 608,420 markers and a total genotyping rate of 0.9854.

## Discussion

The genetic findings of this study could help us to understand the genetic pattern of our population and design association studies. The results of the TLGS indicated that the prevalence of cardiometabolic risk factors is speedily increasing in our population, necessitating further investigations for genetic background information to study the interactions between environmental and genetic factors. The incidence of NCDs, especially cardiometabolic diseases, is increasing worldwide due to changes in lifestyle [18], and various countries have investigated their populations using genetic dissection to understand the genetic pattern of these complex disorders. Unfortunately, established health education and lifestyle interventions in American and European populations cannot be implemented in other regions of the world (such as Iran) due to differences in susceptibility, nutrition, social circumstances, and culture.

The TCGS was designed in Iran as a population-based genetic study, and is one of the very first studies of its kind in the Middle East, addressing the knowledge gap on the genetic patterns of the Iranian population. This study will clarify the role of environmental factors in Iran, along with environmental-genetic interactions. Combining the epidemiological findings in TLGS with genetic data has led to the design of Mendelian randomization studies to change some concepts about the interaction of lipid variation and cardiovascular diseases. In addition, this study may clarify missing heritability data and aid in the understanding of NCD-related problems [19].

However, the delineation of shared phenotypes facilitated by GWAS is of great interest, such as the recent insight into the relationship between height and cardiovascular disease [20]. Results of this study will provide a comprehensive understanding of personalized genomic data and could be used to help to governmental organizations use genetic knowledge to promote personalized health care that facilitates the prevention and management of NCDs.

### Strengths and Weaknesses

This study was based on a national project that selected a representative sample of residents in district-13 of Tehran, the capital of Iran. The project included detailed information on individuals, households, and family relationships (along with environmental, biomedical, and biochemical factors that could be linked to the rate of NCDs) to create a cohort of the entire population residing in Tehran. We recently reviewed the literature in this field, and to our knowledge the present study is the first population-based cohort study for cardiometabolic genetic risk factors in Iran.

In the TCGS, data have been thoroughly and robustly collected on a wide range of clinical parameters, focusing on quantitative traits that are well established as risk factors for cardiometabolic disease. This cohort includes substantial numbers that represent the full adult spectrum of ages, lifestyles, and demography, and includes important phenotypes and quantitative traits to allow population-based genetic and epidemiological research on many important diseases/risks related to NCDs. The family-based approach of the TCGS enables the study to take a flexible approach to gene discovery, encompassing association and linkage approaches, and gives the potential to study aspects such as heritability of disease-related traits and parent-of-origin effects. The combination of linkage and association approaches has proven very effective in studies of various diseases/disease traits [21].

Data in this study can be linked anonymously to routinely collected samples from different cohorts in Iran. This linkage effectively converts this longitudinal study into a national study, with pluripotential outcomes. The TCGS can contribute as a major partner to GWAS meta-analysis consortia, enabling the study of SNPs of low minor allele frequency (1-10%). The TCGS is one of the largest family-based genetic epidemiology studies in Iran and the Middle East.

Although the family-based nature of the cohort is an important strength of this study, providing an efficient strategy for DNA sequence-based studies, relatedness of cohort members will be a confounding factor in some analyses, and may require statistical adjustment. The other weakness of the study is related to the Tehranian nature of the population, rather than a comprehensive Iranian cohort.

## Appendix

Multimedia Appendix 1 Phases of the Tehran Lipid and Glucose Study (duration and number of participants) from 1999 to 2014. Horizontal arrows show the number of people entering and exiting in each phase. Vertical lines show the number of the individuals moving between phases.

Multimedia Appendix 2 Demographic and laboratory information for children and young Tehran Cardiometabolic Genetic Study participants.

Multimedia Appendix 3 Demographic and laboratory information for adults and the elderly among Tehran Cardiometabolic Genetic Study participants.

Multimedia Appendix 4 Phenotype frequency among adult Tehran Cardiometabolic Genetic Study participants.

Multimedia Appendix 5 Genotype failure rate versus heterozygosity across all individuals in the study. The vertical dashed line shows all individuals with a genotype failure rate >0.03 who were excluded, and the horizontal dashed lines represent a heterozygosity rate 3 standard deviations from the mean.

Multimedia Appendix 6 PCAAncestry clustering based on genome-wide association data.

Multimedia Appendix 7 Histogram of missing data rate across all individuals passing per-individual quality control. SNP: single-nucleotide polymorphism; K: thousand.

Multimedia Appendix 8 Peer review report of the grant reviewers.

## Abbreviations

CpG: cytosine phosphate guanine
DNA: deoxyribonucleic acid
ERC: Endocrine Research Center
GWAS: genome-wide association study
IBD: identity by descent
NCD: noncommunicable disease
RIES: Research Institute for Endocrine Sciences
SNP: single-nucleotide polymorphism
TCGS: Tehran Cardiometabolic Genetic Study
TLGS: Tehran Lipid and Glucose Study
