# Supplementary figures and images for: Rationale and Design of a Genetic Study on Cardiometabolic Risk Factors: Protocol for the Tehran Cardiometabolic Genetic Study (TCGS)
Source: JMIR Res Protoc. 2017 Feb 23;6(2):e28. doi: 10.2196/resprot.6050 (PMC5344981; doi:10.2196/resprot.6050)

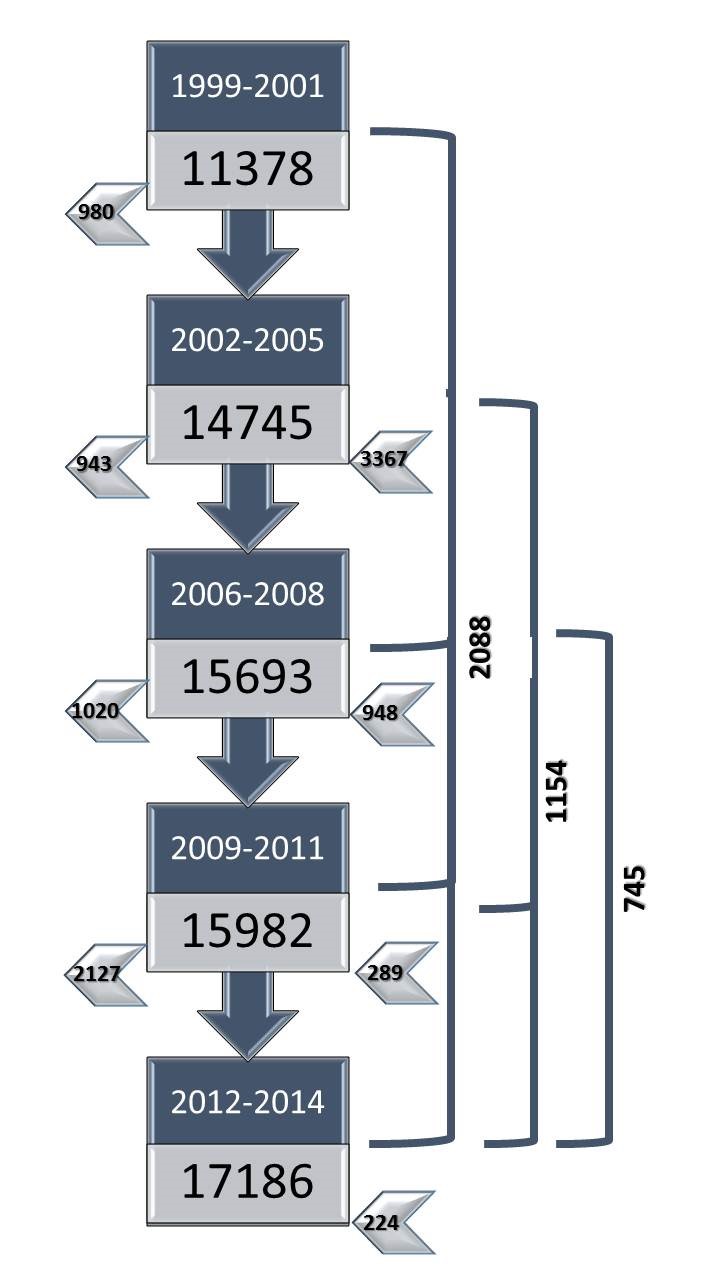

Supplement: Multimedia Appendix 1 [file resprot_v6i2e28_app1.jpg]

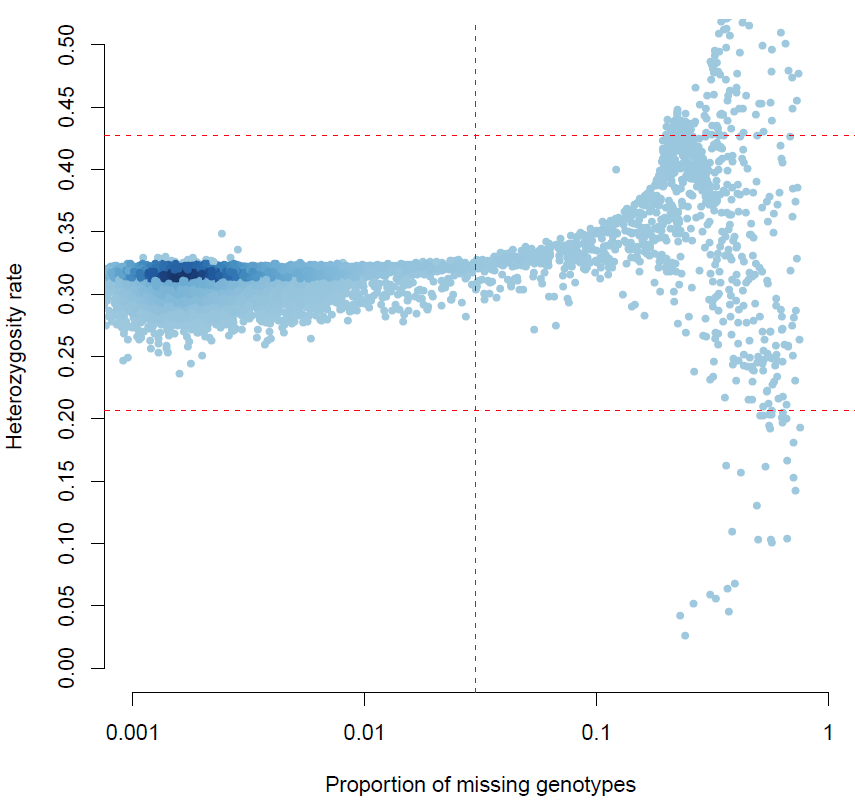

Supplement: Multimedia Appendix 5 [file resprot_v6i2e28_app5.png]

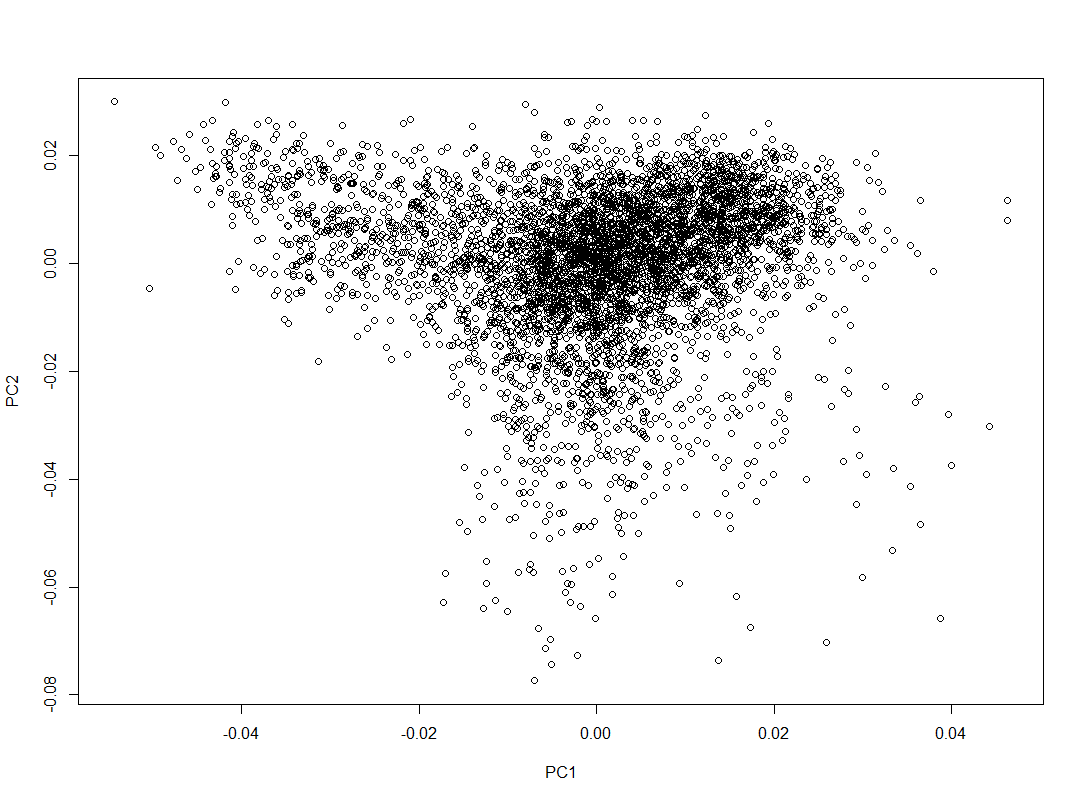

Supplement: Multimedia Appendix 6 [file resprot_v6i2e28_app6.png]

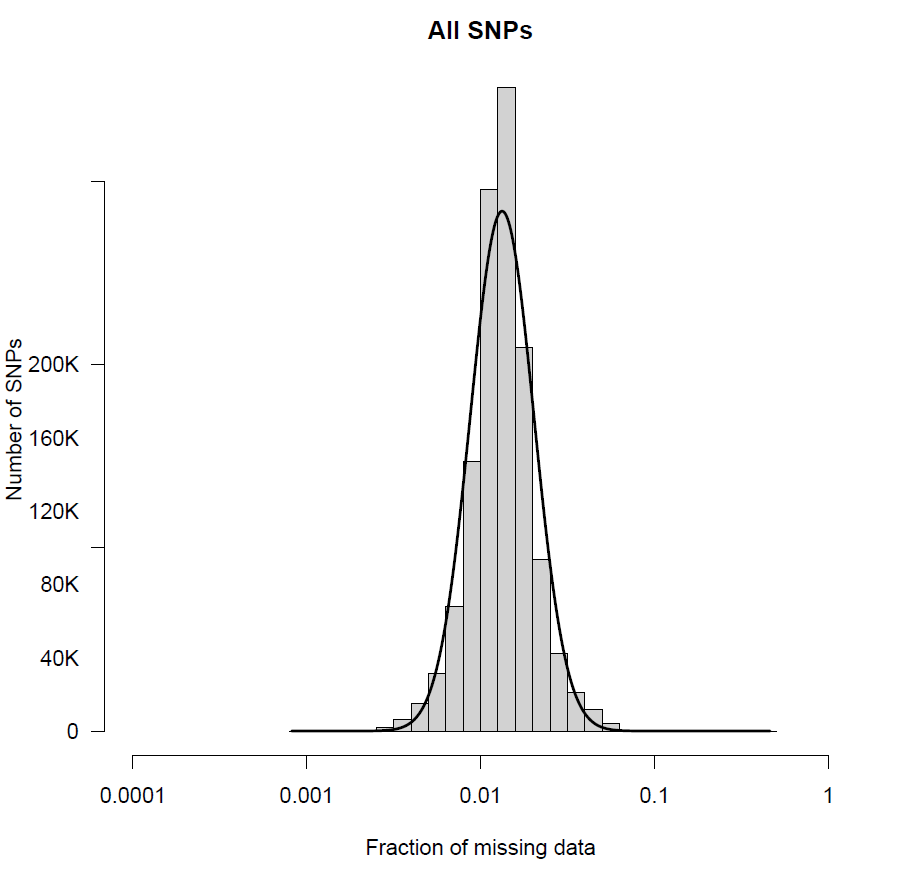

Supplement: Multimedia Appendix 7 [file resprot_v6i2e28_app7.png]
