# Supplementary material for: Rationale and Design of a Genetic Study on Cardiometabolic Risk Factors: Protocol for the Tehran Cardiometabolic Genetic Study (TCGS)
Source: JMIR Res Protoc. 2017 Feb 23;6(2):e28. doi: 10.2196/resprot.6050 (PMC5344981; doi:10.2196/resprot.6050)
Supplement: Multimedia Appendix 2 [file resprot_v6i2e28_app2.pdf]

Multimedia Appendix 1: Table 1. Demographic and laboratory information for child and young TCGS participants

Descriptive	< 10 yrs.					10-18 yrs.					19-30 yrs.				
	Baseline	2002-2005	2006-2008	2009-2011	2012-2014	Baseline	2002-2005	2006-2008	2009-2011	2012-2014	Baseline	2002-2005	2006-2008	2009-2011	2012-2014
**Number**	1121	960	841	623	426	2548	1676	1442	1141	944	2366	2417	2580	2775	2268
**Follow-up time (Median)**	0	4	7	6	6	0	7	10	10	13	0	7	10	10	13
**Age (Years)**	6.4 (1.9)	6.9 (2.2)	7.1 (2.1)	7.4 (2.2)	7.3 (2.1)	14.1 (2.5)	14.8 (2.2)	14.8 (2.3)	14.6 (2.2)	14.6 (2.3)	24.3 (3.6)	24.1 (3.5)	24.3 (3.3)	24.9 (3.3)	25.3 (3.4)
**Never smokers**	1121	-	-	-	-	1134	-	-	-	-	2133	2092	2296	2429	1916
**Marital status n (%)**															
**Single/never married**	1121 (100)	960 (100)	841 (100)	623.0	425 (99.8)	2529 (99.3)	1660 (99)	1437 (99.6)	1134 (99.4)	937 (99.3)	1253 (53)	1367 (56.6)	1523 (59)	1672 (60.3)	1409 (62.1)
**Married**	-	-	-	-	1 (99.8)	19 (0.7)	15 (0.9)	5 (0.4)	4 (0.4)	6 (0.6)	1102 (46.6)	1039 (43)	1099 (42.6)	1074 (38.7)	836 (36.9)
**Divorced**	-	-	-	-	-	-	-	-	-	-	11 (0.5)	9 (0.4)	12 (0.5)	23 (0.8)	20 (0.9)
**Widowed**	-	-	-	-	-	-	-	-	-	-	-	1 (0)	1 (0)	2 (0.1)	-
**Education and work n (%)**															
**Literate**	593 (52.9)	547 (57)	516 (61.4)	425 (68.2)	283 (66.4)	2541 (99.7)	1674 (99.9)	1416 (98.2)	1140 (99.9)	943 (99.9)	2361 (99.8)	2413 (99.8)	2592 (100.5)	2769 (99.8)	2266 (99.9)
**Employed**	2 (0.2)	-	-	-	-	1148 (45.1)	82 (4.9)	63 (4.5)	17 (1.5)	9 (1)	1377 (58.2)	926 (38.3)	927 (35.9)	784 (28.3)	527 (23.2)
**Anthropometrics mean (SD)**															
**Height (cm)**	116 (12)	121 (15)	123 (14)	124 (14)	125 (14)	156 (13)	160 (11)	161 (11)	161 (11)	161 (11)	165 (9)	166 (10)	166 (10)	166 (10)	168 (10)
**Weight (kg)**	21 (7)	25 (10)	26 (12)	27 (11)	28 (11)	50 (15)	55 (15)	57 (16)	58 (16)	58 (17)	66 (14)	67 (15)	68 (16)	70 (16)	71 (17)
**BMI, kg/m^2**	16 (3)	16 (3)	17 (6)	17 (6)	17 (5)	20 (4)	21 (4)	22 (5)	22 (5)	22 (5)	24 (4)	25 (5)	24 (5)	25 (5)	25 (5)
**Waist circumference (cm)**	54 (6)	58 (9)	58 (10)	60 (10)	60 (11)	69 (11)	74 (11)	74 (12)	78 (12)	77 (13)	79 (11)	82 (12)	81 (13)	86 (12)	86 (13)
**Hip circumference (cm)**	63 (7)	67 (10)	67 (10)	68 (9)	68 (10)	86 (12)	90 (10)	91 (10)	91 (11)	90 (11)	98 (9)	98 (9)	98 (8)	98 (9)	98 (9)
**Wrist circumference (cm)**	12.5 (1)	13 (1.3)	13 (1.4)	13.2 (1.3)	12.9 (1.4)	15.3 (1.4)	15.7 (1.4)	15.6 (1.5)	15.7 (1.4)	15.3 (1.4)	16.1 (1.3)	16.2 (1.5)	15.9 (1.5)	16.1 (1.7)	15.7 (1.6)
**Systolic blood pressure (mmHg)**	102 (12)	92 (11)	93 (12)	96 (13)	95 (13)	105 (12)	102 (12)	101 (12)	102 (12)	103 (12)	110 (11)	107 (12)	105 (12)	107 (13)	107 (12)
**Diastolic blood pressure**	70 (10)	62 (9)	61 (9)	61 (12)	62 (12)	71 (9)	68 (9)	66 (9)	68 (10)	69 (10)	73 (9)	70 (9)	69 (9)	72 (9)	72 (9)
**Laboratory Values mean (SD)**															
**Fasting glucose (mg/dl)**	84 (9)	86 (8)	85 (8)	90 (7)	91 (8)	89 (8)	88 (11)	87 (8)	93 (7)	93 (7)	86 (10)	86 (10)	85 (9)	89 (14)	90 (11)
**Total cholesterol (mg/dl)**	171 (30)	163 (28)	161 (28)	164 (30)	164 (27)	168 (32)	155 (29)	154 (30)	153 (28)	157 (29)	180 (37)	166 (34)	165 (33)	166 (33)	172 (35)
**LDL_C (mg/dl)**	107 (27)	101 (26)	97 (25)	94 (26)	92 (25)	103 (29)	94 (25)	90 (26)	85 (24)	86 (25)	112 (32)	103 (29)	100 (28)	96 (29)	100 (31)
**HDL_C l (mg/dl)**	46 (11)	45 (11)	47 (12)	54 (12)	56 (12)	43 (10)	40 (10)	43 (10)	50 (11)	51 (12)	43 (11)	40 (10)	43 (10)	49 (11)	50 (12)
**Non HDL cholesterol (mg/dl)**	125 (29)	119 (28)	114 (27)	110 (29)	109 (28)	125 (33)	115 (29)	110 (29)	104 (28)	106 (29)	136 (38)	125 (34)	122 (34)	117 (34)	122 (36)
**Triglycerides (mg/dl)**	90 (38)	88 (39)	85 (38)	81 (40)	82 (40)	110 (59)	104 (53)	102 (55)	95 (49)	101 (53)	123 (87)	113 (70)	111 (72)	106 (92)	112 (72)
**Using drug (%)**															
**Lipid**	-	-	-	2 (0.3)	1 (0.2)	-	1 (0.1)	7 (0.5)	3 (0.3)	3 (0.3)	4 (0.2)	7 (0.3)	16 (0.6)	7 (0.3)	7 (0.3)
**hypertension**	-	-	-	-	-	1 (0)	1 (0.1)	-	-	2 (0.2)	5 (0.2)	5 (71.4)	5 (0.2)	8 (0.3)	9 (0.4)
**Diabetes**	-	-	-	-	1 (0.2)	2 (0.1)	2 (0.1)	1 (0.1)	2 (0.2)	1 (0.1)	3 (0.1)	8 (0.3)	9 (0.3)	24 (0.9)	22 (1)
