# Supplementary material for: Rationale and Design of a Genetic Study on Cardiometabolic Risk Factors: Protocol for the Tehran Cardiometabolic Genetic Study (TCGS)
Source: JMIR Res Protoc. 2017 Feb 23;6(2):e28. doi: 10.2196/resprot.6050 (PMC5344981; doi:10.2196/resprot.6050)
Supplement: Multimedia Appendix 3 [file resprot_v6i2e28_app3.pdf]

Multimedia Appendix 2: Table 2. Demographic and laboratory information for adult elderly among TCGS

Descriptive	31-50 yrs.					51-70 yrs.					More than 71 yrs.				
	Baseline	2002-2005	2006-2008	2009-2011	2012-2014	Baseline	2002-2005	2006-2008	2009-2011	2012-2014	Baseline	2002-2005	2006-2008	2009-2011	2012-2014
**Number**	3812	3803	4048	4327	4332	2245	2443	2684	2981	3102	266	423	587	890	1044
**Follow-up time (Median)**	0	7	10	13	13	0	7	10	13	16	0	7	10	13	16
**Age (Years)**	39.7 (5.7)	40.1 (5.5)	40.4 (5.5)	40.6 (5.7)	40.5 (5.9)	59.1 (5.5)	59.5 (5.7)	59.4 (5.7)	59.5 (5.7)	59.4 (5.6)	74.4 (3.2)	75.1 (3.8)	75.3 (3.9)	76.4 (4.5)	77 (4.9)
**Never smokers**	3103	3099	3425	3711	3761	1963	2169	2372	2648	2721	240	388	538	834	976
**Marital status n (%)**															
**Single/never married**	210 (5.5)	239 (6.3)	274 (6.8)	351 (8.1)	409 (9.4)	9 (0.4)	17 (0.7)	22 (0.8)	32 (1.1)	48 (1.5)	-	1 (0.2)	1 (0.2)	3 (0.3)	4 (0.4)
**Married**	3480 (91.3)	3436 (90.3)	3712 (91.7)	3852 (89)	3783 (87.3)	1975 (88)	2090 (85.6)	2289 (85.3)	2549 (85.5)	2683 (86.5)	191 (71.8)	301 (71.2)	424 (72.2)	609 (68.4)	709 (67.9)
**Divorced**	47 (1.2)	63 (1.7)	67 (1.7)	66 (1.5)	90 (2.1)	12 (0.5)	14 (0.6)	34 (1.3)	43 (1.4)	53 (1.7)	3 (1.1)	4 (0.9)	10 (1.7)	9 (1)	8 (1.1)
**Widowed**	75 (2)	63 (1.7)	51 (1.3)	55 (1.3)	47 (1.1)	249 (11.1)	322 (13.2)	358 (13.3)	356 (11.9)	316 (10.2)	72 (27.1)	116 (27.4)	154 (26.2)	269 (30.2)	323 (30.9)
**Education and work n (%)**															
**Literate**	3726 (97.7)	3749 (98.6)	4017 (99.2)	4310 (99.6)	4320 (99.7)	1707 (76)	1982 (81.1)	2300 (85.7)	2727 (91.5)	2949 (95.1)	141 (53)	252 (59.6)	359 (61.2)	578 (64.9)	767 (73.5)
**Employed**	1873 (49.1)	1885 (49.6)	1933 (47.8)	1940 (44.8)	1858 (42.9)	620 (27.6)	1839 (75.3)	1970 (73.4)	2218 (74.4)	2297 (74)	42 (15.8)	363 (85.8)	502 (85.5)	814 (91.5)	965 (92.4)
**Anthropometrics mean (SD)**															
**Height (cm)**	163 (9)	163 (9)	163 (9)	164 (10)	165 (10)	160 (9)	160 (9)	160 (9)	160 (10)	160 (10)	159 (9)	160 (9)	159 (9)	159 (10)	159 (10)
**Weight (kg)**	72 (13)	74 (13)	75 (14)	76 (14)	77 (15)	71 (12)	73 (12)	73 (12)	74 (12)	75 (13)	66 (11)	68 (11)	68 (11)	69 (11)	69 (12)
**BMI, kg/m²**	27 (5)	28 (5)	28 (5)	28 (5)	28 (5)	28 (5)	29 (5)	29 (5)	29 (5)	29 (5)	26 (4)	26 (4)	27 (5)	27 (5)	27 (5)
**Waist circumference (cm)**	89 (11)	92 (11)	91 (12)	94 (11)	93 (12)	94 (11)	97 (11)	97 (11)	99 (11)	98 (11)	92 (11)	96 (10)	96 (11)	98 (11)	97 (11)
**Hip circumference (cm)**	102 (9)	103 (9)	103 (9)	102 (8)	101 (8)	101 (10)	103 (9)	103 (10)	101 (8)	101 (9)	97 (8)	98 (8)	99 (9)	98 (8)	97 (8)
**Wrist circumference (cm)**	16.7 (1.3)	16.9 (1.4)	16.7 (1.5)	16.7 (1.5)	16.2 (1.6)	17.2 (1.2)	17.3 (1.3)	17.1 (1.4)	17.1 (1.4)	16.7 (1.5)	17.2 (1.2)	17.5 (1.3)	17.3 (1.3)	17.4 (1.3)	17 (1.5)
**Systolic blood pressure (mmHg)**	115 (15)	112 (14)	110 (14)	112 (14)	112 (14)	132 (22)	128 (20)	126 (20)	126 (20)	125 (19)	142 (22)	138 (23)	137 (22)	136 (23)	135 (22)
**Diastolic blood pressure**	78 (10)	75 (10)	74 (10)	77 (10)	77 (10)	82 (12)	79 (11)	77 (11)	81 (11)	80 (10)	80 (13)	76 (12)	74 (11)	78 (12)	78 (12)
**Laboratory Values mean (SD)**															
**Fasting glucose (mg/dl)**	95 (26)	95 (25)	93 (24)	98 (25)	97 (24)	112 (47)	111 (43)	109 (42)	114 (42)	112 (38)	108 (40)	110 (38)	108 (36)	114 (42)	112 (38)
**Total cholesterol (mg/dl)**	209 (42)	192 (38)	190 (37)	189 (36)	191 (36)	233 (48)	212 (42)	207 (41)	202 (43)	200 (41)	225 (48)	201 (38)	197 (40)	195 (42)	193 (42)
**LDL_C (mg/dl)**	133 (35)	121 (33)	118 (32)	114 (31)	113 (31)	152 (40)	136 (36)	130 (35)	121 (36)	119 (37)	148 (40)	131 (32)	125 (35)	118 (37)	115 (37)
**HDL_C (mg/dl)**	41 (11)	38 (10)	41 (10)	47 (11)	48 (12)	43 (11)	39 (10)	42 (10)	47 (11)	49 (12)	43 (10)	39 (10)	42 (10)	47 (11)	50 (12)
**Non HDL_C (mg/dl)**	167 (42)	153 (38)	149 (37)	142 (37)	142 (37)	190 (47)	172 (41)	165 (40)	154 (41)	151 (41)	182 (47)	162 (37)	155 (39)	147 (41)	143 (41)
**Triglycerides mg/dl**	179 (123)	167 (111)	162 (109)	148 (100)	151 (113)	200 (127)	188 (112)	179 (100)	168 (104)	163 (90)	180 (107)	160 (97)	154 (78)	146 (70)	143 (67)
**Using drug**															
**Lipid**	43 (1.1)	80 (2.1)	98 (2.4)	167 (3.9)	197 (4.5)	116 (5.2)	192 (7.9)	320 (11.9)	544 (18.2)	760 (24.5)	17 (6.4)	23 (5.4)	56 (9.5)	186 (20.9)	285 (27.3)
**Hypertension**	8 (0.2)	132 (3.5)	72 (1.8)	139 (3.2)	164 (3.8)	11 (0.5)	442 (18.1)	307 (11.4)	798 (26.8)	887 (28.6)	-	115 (27.2)	80 (13.6)	335 (37.6)	501 (48)
**Diabetes**	59 (1.5)	113 (3)	122 (3)	152 (3.5)	170 (3.9)	202 (9)	296 (12.1)	362 (13.5)	460 (15.4)	550 (17.7)	28 (10.5)	53 (12.5)	78 (13.3)	170 (19.1)	219 (21)
