# Supplementary material for: Rationale and Design of a Genetic Study on Cardiometabolic Risk Factors: Protocol for the Tehran Cardiometabolic Genetic Study (TCGS)
Source: JMIR Res Protoc. 2017 Feb 23;6(2):e28. doi: 10.2196/resprot.6050 (PMC5344981; doi:10.2196/resprot.6050)
Supplement: Multimedia Appendix 4 [file resprot_v6i2e28_app4.pdf]

Multimedia Appendix 3: Table 3. Phenotype frequency among adult TCGS participants

Descriptive	19-30 yrs.					31-50 yrs.				
	Baseline	2002-2005	2006-2008	2009-2011	2012-2014	Baseline	2002-2005	2006-2008	2009-2011	2012-2014
**Number**	2366	2417	2580	2775	2268	3812	3803	4048	4327	4332
**Hypertension status**										
**Normal**	1514 (64)	1762 (72.9)	2008 (77.8)	1929 (69.5)	1546 (68.2)	1882 (49.4)	2239 (58.9)	2539 (62.7)	2310 (53.4)	2249 (51.9)
**hypertension**	83 (3.5)	69 (2.9)	54 (2.1)	113 (4.1)	93 (4.1)	513 (13.5)	349 (9.2)	304 (7.5)	593 (13.7)	505 (11.7)
**Pre-hypertension**	708 (29.9)	511 (21.1)	490 (19)	688 (24.8)	597 (26.3)	1339 (35.1)	1100 (28.9)	1161 (28.7)	1373 (31.7)	1541 (35.6)
**Dyslipidemia status**										
**Normal**	816 (34.5)	597 (24.7)	808 (31.3)	928 (33.4)	698 (30.8)	519 (13.6)	446 (11.7)	589 (14.6)	732 (16.9)	780 (18)
**Dyslipidemia**	310 (13.1)	217 (9)	211 (8.2)	175 (6.3)	172 (7.6)	1119 (29.4)	843 (22.2)	809 (20)	760 (17.6)	744 (17.2)
**Pre-dyslipidemia**	1149 (48.6)	999 (41.3)	1093 (42.4)	1159 (41.8)	1021 (45)	2069 (54.3)	2070 (54.4)	2246 (55.5)	2286 (52.8)	2249 (51.9)
**Diabetes status**										
**Normal**	2093 (88.5)	2168 (89.7)	2434 (94.3)	2477 (89.3)	1974 (87)	2655 (69.6)	2688 (70.7)	3157 (78)	3045 (70.4)	3033 (70)
**Diabetic**	14 (0.6)	17 (0.7)	16 (0.6)	32 (1.2)	32 (1.4)	277 (7.3)	281 (7.4)	279 (6.9)	289 (6.7)	310 (7.2)
**Pre-diabetic**	145 (6.1)	122 (5)	88 (3.4)	213 (7.7)	224 (9.9)	738 (19.4)	681 (17.9)	560 (13.8)	944 (21.8)	955 (22)
**Obesity status**										
**Underweight**	437 (18.5)	345 (14.3)	397 (15.4)	341 (12.3)	255 (11.2)	137 (3.6)	91 (2.4)	87 (2.1)	78 (1.8)	83 (1.9)
**Normal**	997 (42.1)	947 (39.2)	1121 (43.4)	1142 (41.2)	903 (39.8)	990 (26)	841 (22.1)	920 (22.7)	945 (21.8)	987 (22.8)
**Obese**	626 (26.5)	636 (26.3)	702 (27.2)	827 (29.8)	709 (31.3)	1654 (43.4)	1569 (41.3)	1853 (45.8)	1972 (45.6)	1882 (43.4)
**Morbid obese**	233 (9.8)	259 (10.7)	286 (11.1)	383 (13.8)	317 (14)	950 (24.9)	1036 (27.2)	1134 (28)	1251 (28.9)	1258 (29)
**Metabolic syndrome status (Mets)**										
**Normal**	1984 (83.9)	1971 (81.5)	2280 (88.4)	2472 (89.1)	2005 (88.4)	2475 (64.9)	2369 (62.3)	2862 (70.7)	3168 (73.2)	3246 (74.9)
**Mets with 3 risk factor**	173 (7.3)	136 (5.6)	142 (5.5)	155 (5.6)	124 (5.5)	738 (19.4)	772 (20.3)	763 (18.8)	715 (16.5)	644 (14.9)
**Mets with 4 risk factor**	26 (1.1)	34 (1.4)	18 (0.7)	44 (1.6)	34 (1.5)	329 (8.6)	284 (7.5)	239 (5.9)	299 (6.9)	249 (5.7)
**Mets with 5 risk factor**	5 (0.2)	4 (0.2)	1 (0)	#VALUE!	4 (0.2)	62 (1.6)	62 (1.6)	45 (1.1)	52 (1.2)	64 (1.5)
	51-70 yrs.					More than 71 yrs.				
**Number**	2245	2443	2684	2981	3102	266	423	587	890	1044
**Hypertension status**										
**Normal**	509 (22.7)	652 (26.7)	795 (29.6)	753 (25.3)	757 (24.4)	39 (14.7)	69 (16.3)	101 (17.2)	134 (15.1)	122 (11.7)
**hypertension**	845 (37.6)	757 (31)	758 (28.2)	1029 (34.5)	997 (32.1)	137 (51.5)	200 (47.3)	257 (43.8)	433 (48.7)	528 (50.6)
**Pre-hypertension**	848 (37.8)	990 (40.5)	1073 (40)	1174 (39.4)	1320 (42.6)	87 (32.7)	145 (34.3)	216 (36.8)	313 (35.2)	386 (37)
**Dyslipidemia status**										
**Normal**	128 (5.7)	144 (5.9)	172 (6.4)	227 (7.6)	231 (7.4)	16 (6)	25 (5.9)	49 (8.3)	49 (5.5)	74 (7.1)
**Dyslipidemia**	1127 (50.2)	1079 (44.2)	1149 (42.8)	1291 (43.3)	1443 (46.5)	123 (46.2)	157 (37.1)	232 (39.5)	421 (47.3)	517 (49.5)
**Pre-dyslipidemia**	954 (42.5)	1092 (44.7)	1259 (46.9)	1298 (43.5)	1266 (40.8)	118 (44.4)	219 (51.8)	274 (46.7)	372 (41.8)	402 (38.5)
**Diabetes status**										
**Normal**	1045 (46.5)	1159 (47.4)	1384 (51.6)	1270 (42.6)	1276 (41.1)	119 (44.7)	172 (40.7)	264 (45)	310 (34.8)	356 (34.1)
**Diabetic**	526 (23.4)	568 (23.3)	605 (22.5)	773 (25.9)	792 (25.5)	66 (24.8)	107 (25.3)	145 (24.7)	268 (30.1)	342 (32.8)
**Pre-diabetic**	617 (27.5)	663 (27.1)	660 (24.6)	919 (30.8)	1013 (32.7)	71 (26.7)	138 (32.6)	171 (29.1)	300 (33.7)	342 (32.8)
**Obesity status**										
**Underweight**	55 (2.4)	47 (1.9)	37 (1.4)	48 (1.6)	46 (1.5)	9 (3.4)	16 (3.8)	28 (4.8)	26 (2.9)	32 (3.1)
**Normal**	519 (23.1)	449 (18.4)	476 (17.7)	454 (15.2)	496 (16)	85 (32)	135 (31.9)	156 (26.6)	203 (22.8)	251 (24)
**Obese**	971 (43.3)	1042 (42.7)	1182 (44)	1238 (41.5)	1266 (40.8)	121 (45.5)	180 (42.6)	260 (44.3)	339 (38.1)	362 (34.7)
**Morbid obese**	667 (29.7)	798 (32.7)	949 (35.4)	1166 (39.1)	1224 (39.5)	40 (15)	73 (17.3)	126 (21.5)	190 (21.3)	232 (22.2)
**Metabolic syndrome status (Mets)**										
**Normal**	1023 (45.6)	1016 (41.6)	1281 (47.7)	1535 (51.5)	1714 (55.3)	135 (50.8)	196 (46.3)	300 (51.1)	433 (48.7)	535 (51.2)
**Mets with 3 risk factor**	530 (23.6)	656 (26.9)	685 (25.5)	687 (23)	717 (23.1)	49 (18.4)	99 (23.4)	128 (21.8)	182 (20.4)	195 (18.7)
**Mets with 4 risk factor**	440 (19.6)	471 (19.3)	454 (16.9)	514 (17.2)	460 (14.8)	56 (21.1)	73 (17.3)	92 (15.7)	101 (11.3)	107 (10.2)
**Mets with 5 risk factor**	161 (7.2)	170 (7)	175 (6.5)	160 (5.4)	128 (4.1)	12 (4.5)	33 (7.8)	36 (6.1)	38 (4.3)	39 (3.7)
