# Supplementary material for: Rationale and Design of a Genetic Study on Cardiometabolic Risk Factors: Protocol for the Tehran Cardiometabolic Genetic Study (TCGS)
Source: JMIR Res Protoc. 2017 Feb 23;6(2):e28. doi: 10.2196/resprot.6050 (PMC5344981; doi:10.2196/resprot.6050)
Supplement: Multimedia Appendix 8 [file resprot_v6i2e28_app8.pdf]

Dear Editor

Herewith the revised version of manuscript ms#6050 entitled “Rationale and design of Genetic study in Cardio-Metabolic risk factors: Tehran Cardio-Metabolic Genetic Study (TCGS)", the response to comments is attached. All the changes in manuscript and supplementary were highlighted.

Reviewer AW:

Major comments

1. The TLGS is described as a community-based program but it would be helpful to include information on how subjects were identified and recruited for the study.

   Response: Agreed and revised. More information about TLGS were added. (P 7-8, last Par)

2. Criteria for identifying outcome events as well as censoring events and/or loss to follow-up should be clarified. Outcome measurement over follow-up is described on p.5 of the supp material, and implies nurses and physicians collected event and death data. However, it is not clear how the study would be notified an event has taken place in order to collect this information, nor is it clear what censoring criteria might be used for various outcomes.

   Response: Agreed and revised. The criteria for various outcome were added to Supp P 6, Par 1.

3. Figure 1 is not very intuitive - a flowchart may be better for describing the cohorts, beginning with TLGS.

   Response: Agreed and revised as suggested

4. Supp Table 1 and 2 (cohort characteristics by age) might be easier to understand if aligned to baseline. Currently the tables seem to show the total number of individuals in each age group, in each study year category (eg. 2002-2005), such that individuals overlap between years - ie. each year set includes subjects in the previous year set as well as those newly entered, minus those who had an event or were lost to follow-up. Perhaps a table displaying characteristics of all participants at baseline would be best, with a variable row for year to indicate how many enrolled in each year (or year category; eg 2002-2005), and a row for follow-up time (median if continuous or categorical, depending on distribution).

   Response: Agreed and revised Table were changed and baseline information were added.

5. More detail regarding GWAs analyses should be given in methods/sup material. Some basic QC information is given, but no description of methods to be performed for assessment of genetic association. Authors may include a few paragraphs outlining the population-based and

family-based analyses that will be conducted as part of this project (at least for major outcomes).

Response: Agreed and revised. Genotyping procedure steps have been transferred to manuscript body and revised in more details. (P13-14)

Minor comments

1. There are many grammatical errors to be fixed prior to publication.
   Response: Agreed and revised.
2. If all individuals participating in this study are of Iranian decent, then it should be stated. If not, and individuals of other ethnicities (living in Tehran) were included, it should be stated or listed in Table 1, and methods accounting for population substructure described in the methods.
   Response: Agreed and revised as suggested. (P 8)

Reviewer AX:

Major comments

1. The description of the cohort is incomplete. Since the cohort is described as a subset from another study (TLGS), more information regarding this study should be provided: eligibility criteria, recruitment, design (family study?), interventions, etc. It is also unclear which part of the

proposed study (TCGS) is separate from TLGS outside from the genetic analyses (specific phenotypes/biomarkers measured? different follow-up?).

What was the frequency of the follow-up assessments? Were the participants selected for TCGS only on the basis of the availability of DNA samples for genetic analyses?

Response: Agreed and revised. (P 9, Par 1)

3. There are some inconsistencies in the numbers mentioned, for example the number of participants in TCGS is 16,247 in the abstract and 16,144 in the Results on page 11. There is a similar issue with the TLGS number (21,216 in the abstract, 20,068 on page 7, 19,905 on page 11). Was TLGS cohort created in 1997 or 1999? Are there plans to genotype more of the TCGS participants (11,497 have been genotyped so far)? A flowchart diagram would help clarify this information (Figure 1 is not intuitive to read).
   Response: Agreed and revised.
4. The analysis plan is not clearly defined. Is the main focus of the study cardiovascular disease and its risk factors?

   Response: The analysis will focus on cardio metabolic risk factors. (P6, Par 2)

5. The protocol regarding the genotyping quality control (p.4-5 of supplementary protocol) is incomplete:

   a) Was the sex verified with the genetic data (“sex-check”)?

   b) Was ethnicity verified with the genetic data (“ethnicity-check”)?

   c) What was the threshold to exclude individuals based on IBS? Was the goal to exclude duplicates? Many individuals seem to be first-degree relatives (pedigrees), I assume those were kept?

   d) What is the meaning of “imputation of individuals with outlying missing genotype”?

   e) Were variants filtered based on deviance form Hardy-Weinberg equilibrium?

   f) The complete QC process should be summarized in the main manuscript with the final number of participants and variants remaining for analysis.

Response: All items have been revised in Genotype Quality Control (QC) section. (P 13-14)

6. The protocol regarding the genetic analyses is incomplete:

a) What are the outcomes of interest?

> **Response:** Agreed and revised. The interested outcomes for analysis were added. (P 10, Par 1)

b) How will relatedness be taken into consideration in the analyses?

> **Response:** Agreed and revised. Average IBS/IBD values have been calculated for each pair of individuals in the present GWAS data set with PLINK program (Supp. P 5, Par 1)

c) How will population stratification be corrected for (sample divided by ethnicity? adjustment for principal components?)

> **Response:** Agreed and revised. Component analysis was done and the result were reported

d) What other covariable will be included in the association analyses?

Response: Education level, physical activity, smoking habits, nutritional habits and drug use will be use as a covariable (P 10)

e) Will imputation based on a reference panel be performed to obtain information about a higher number of variants?

> **Response**: Yes, the reference panel will be designed. (P 10)

f) The epigenetic analysis part (p. 9) requires more explanations (only appears in this paragraph). Will specific genes be targeted? How will they be selected? What method will be used for the Epigenome-wide analysis?

Response: Agreed and revised. (P11)

g) What methods will be used for the gene-gene and gene-environment interactions analyses (only mentioned in the abstract and in the conclusion).

Response: Agreed and revised. (P11)

h) Mendelian randomization is only mentioned in the Conclusion on page 13 without more explanation on whether this approach will be used in the cohort.

Response: Agreed and revised. (P 10, Par 1)

7. The introduction should focus more on cardio-metabolic diseases as it seems to be the main focus of the study.

   Response: Agreed and revised.

8. The primary objectives should be better specified (for example “diseases common in the population” is very broad). The secondary objectives are unclear: heritability study?, comparison between which groups? What is the meaning of “pattern of genetic risk factors”?

   Response: Agreed and revised. (P6, Par 2)

9. Did the participants provide consent for the genetic analyses?

   Response: Yes, it is mention in page 8 Par 1

10. The manuscript should be revised regarding English language.

    Response: The manuscript was revised

Minor comments

1. A reference to the supplementary protocol should come earlier and be repeated in the methods and results sections.

   Response: Agreed and revised. The first time were referred to the supplementary in page 13 and then in p14-15.

2. The “Drawing family tree” section should be moved further as it is based on the genotyping data. Also, the letter B) is used twice.

   Response: Agreed and revised as suggested.

2. Different units are used in the Supplementary protocol (e.g. mg/dL vs mmol/L for cholesterol).

Response: Agreed and revised.

4. The dyslipidemia paragraph in the Supplementary protocol contains information on hypertension and diabetes that appears out of place.

Response: Agreed and revised. Unrelated topics were removed.

5. Legends should be added to the supplementary tables, for examples stating what the numbers represent (n - % vs mean +/- SD, etc).
